# The Differential Diagnosis between Yellow Fever, Dengue Fever, Malarial Fever, and Acute Yellow Atrophy of the Liver*Read before the Bexar County Medical Society.

**Published:** 1904-01

**Authors:** F. Paschal

**Affiliations:** San Antonio, Texas


For Texas Medical Journal.

### The Differential Diagnosis Between Yellow Fever, Dengue Fever, Malarial Fever, and Acute Yellow Atrophy of the Liver.*

BY F. PASCHAL, M. D., SAN ANTONIO, TEXAS.

Yellow fever, dengue fever, pernicious malarial fever, and acute yellow atrophy of the liver have some symptoms so much alike as to require a careful study to differentiate them, but, with the possible exception of dengue fever, their resemblance to yellow fever is distinct, especially so in their etiology, combined symptomatology, and pathology.

In order to deal intelligently with the differential diagnosis of these diseases, the etiology, and pathology of those that are known, must be considered, and also a somewhat detailed symptomatology of yellow fever must be given.

Yellow fever is an acute specific infectious disease, manifesting itself in a mild, a moderately severe, or a severe form; not only does the severity of individual cases differ according to the intensity of the infection, personal condition, and environment, but epidemics vary. The etiology of yellow fever does not seem to be definitely determined. The bacillus icteroides of Sanarelli was at one time thought possibly to be the cause of the disease, but the work of the commission organized in 1900 for the purpose of studying yellow fever in Cuba, endeavored to isolate the bacillus icteroides of Sanarelli, but in twenty-four carefully studied cases the results were negative. Moreover, the careful study of eleven autopsies was equally barren as to the presence of any particular micro-organism.

*Read before the Bexar County Medical Society.

This was also true of fresh and stained specimens of blood. Although a microscopic and bacteriologic study of the blood was negative, the infective agent must, nevertheless, be present in the blood, for the disease was produced by injecting small quantities of blood into non-immunes. The infective agent was present at least during the first, second, and third day of the fever. The disease was also produced by injecting a small quantity of bacteria free serum filtrate. Thoroughly conducted investigations prove conclusively that the disease is conveyed by an intermediate host, the stegomyia fasciata. Furthermore, infected mosquitoes for periods varying from thirty-nine to fifty-seven days after contamination, are capable of conveying the disease. Infected stegomyiae may survive for a period of seventy-one days. This is important, owing to the relation they bear to the propagation of the disease.

Yellow fever usually commences suddenly, and is manifested by a well marked rigor, or only a sense of chilliness and discomfort. The fever rises rapidly and reaches its acme in a few hours from the onset. There are pains in the forehead, the eyes ache, the muscles in the back, loins, thighs, calves, often ache severely, even in mild cases. The face is turbid and not infrequently of dusky red hue. The conjunctivæ are congested, and shiny, with a yellow tinge, the eyes are sometimes red and sensitive to light. The jaundice becomes more distinct after the first or second day, the skin showing the same combination of capillary stasis with an icteroid hue, as the eyes. The skin may be either dry or bathed in a copious perspiration. Following the chill, there is sometimes an abnormal coldness of the surface, while rectal thermometry shows a marked rise in the temperature. The temperature rises rapidly after the chill to 103° to 104°, though it is said that the initial rise has been as great as 110°; this is considered phenomenal. At the end of the third day the maximum fever will have been attained, which is rarely more than 104° to 105°. Between this period and the fourth day of the disease, slight variations, hardly amounting to distinct remission; on the fourth day there is a rapid defervesence; it is not an intermission, but a remission, for the temperature only falls to 100° or 101°. The period of remission lasts from a few hours to two or three days after which the second rise begins, one that does not take place quite as rapidly as the first, and is not usually preceded by a chill or rigors, and a temperature of 104° to 105° is sometimes reached. The temperature now remains stationary from one to two days; it then falls to normal and remains so. The range of temperature divides this disease into three parts, first, the stage of invasion, the febrile stage, or period of exacerbation, second the stage of remission, calm or passive stage; third stage, second exacerbation or collapse. The temperature may not be as typical as described, but even in the variations of the temperature curve there is a family resemblance difficult to confound with the clinical curves of any other acute febrile infectious disease.

The pulse does not increase in frequency with the rise of temperature, there being no correlation between the temperature and the pulse. It rarely exceeds 110°, and frequently falls as low as 30° or 40°. The lack of correlation is exhibited best during the first three days of the fever.

The fall of the pulse is most particularly a symptom of the first three days, and a lack of correlation between the pulse and temperature is what constitutes the well known Faget's law.

The tongue is covered at the outset with a thick, yellowish coating, except at the edges, which remain red. It is often pointed, and, as the disease advances, may become dry, brown, cracked and fissured.

The buccal mucous membrane is bright red at first, subsequently becoming œdematous. The gums are swollen and bleed on pressure. Sordes form on the teeth.

The bowels are usually constipated, but when diarrhœa does occur fluid blood is apt to be mingled with the discharges.

Immediately following the chill nausea and vomiting are present. First the contents of the stomach are voided, then a yellowish green matter. In severe cases the characteristic black vomit is present, the result of hemorrhage into the stomach. There are hemorrhagic extravasations from the mucous membranes of different organs.

The urine is scanty; there is a decided diminution in the excretion of solids. Albumen is found so constantly as to be generally regarded as one of the pathognomonic symptoms. It usually appears within seventy-two hours; there may be only a trace found by careful testing of the evening urine. In the second stage, the amount of albumen may be large, and granular and other casts are found, and there is a decided tendency to uremia.

Pain is quite severe over the lumbar and epigastric regions, which are quite sensitive to pressure. Convulsive twitchings of the muscles and diaphragmatic contractions, are often present before death.

The pathological changes of yellow fever have much that is common to contagious and malarial diseases. Its most constant and characteristic lesion is found in the liver. The liver is slightly enlarged; it may, however, be normal or even slightly diminished in size. Its most striking changes is in its color, which may vary from lemon-yellow to dark brown. It is bloodless, fatty, degenerated, and friable. The gall bladder is contracted, usually empty, but sometimes containing a thick tarry fluid.

The spleen is usually normal in size and color, though sometimes found congested and slightly increased in size.

The stomach and intestines are congested and eroded.

The heart flabby, fatty degenerated, the muscles softened.

The kidneys are congested and show signs of parenchymatous inflammation. The other tissues present a marked icteroid hue.

An analysis of the symptoms shows that they are present in several acute infectious diseases, and reliance to differentiate them can only be placed, first, upon a prevailing epidemic at a given place, or some other place even remotely from it; second, by its being a fever of one paroxysm, with lack of correlation between the pulse and the temperature, by the presence of albumen in the urine, jaundice, black vomit, suppression of urine, and the general tendency to hemorrhages from mucous surfaces; third, by the post-mortem findings.

Dengue can only be differentiated from yellow fever, first, by the correlation between the pulse and the temperature; second, the absence of albumen in the urine; third, absence of jaundice; fourth, absence of black vomit; fifth, its being non-fatal; sixth, presence of an eruption.

In the absence of a universally recognized specific germ of yellow fever, it is impossible to differentiate, positively, between yellow fever and dengue. They both follow the same geographical distribution, except that dengue has prevailed in Asia and Egypt, where yellow fever is unknown.

The two diseases are found to exist at the same time and place, and the disease taken for dengue has frequently been found to have albumen in the urine, jaundice, and black vomit, and a lack of correlation between the pulse and temperature.

Laying aside the classical description of yellow fever, and considering the mild forms of this disease, how does it differ from dengue?

It is very difficult to differentiate between the mild form of yellow fever and dengue. They follow the same epidemical course at the same seasons of the year, in almost identical latitudes, and have as an intermediate host the mosquito, and both cease to exist as soon as a freeze inhibits their immediate hosts, the mosquitoes.

If an epidemic is present of a fever that is causing no deaths, it is just as well, perhaps, to give it a commercial diagnosis and call it dengue. It must be left for future study to determine whether places which have been visited by dengue fever will be found to be fertile soil when visited by yellow fever; in other words, whether immunity is conferred by dengue from yellow fever upon a large percentage of the population which has known an epidemic of dengue in previous years.

The differential diagnosis between yellow fever and pernicious (an unfortunate adjective attached to malarial fever) may be differentiated from yellow fever by first, the disease being rarely seen in temperate climates; second, that it never assumes an epidemic or endemic form; that it manifests a periodicity; fourth, that it depends invariably upon the æstivo-autumnal parasite, or malarial plasmodium.

The microscope will always demonstrate the malarial parasite in all cases of malarial fever, and certainly differentiate it from yellow fever.

It is true that the plasmodium has been found to be present in cases of yellow fever, but is so rare that it should not preclude the possibility of yellow fever during the prevalence of this disease.

The type of malarial fever is as varied as the different forms in which it manifests itself, and can be easily differentiated from yellow fever by the microscope.

The differential diagnosis between yellow fever and acute yellow atrophy of the liver can be determined as given by Loomis as follows: "First, in acute yellow atrophy of the liver, the liver is diminishing in size from day to day, while in yellow fever it is steadily increasing; second, the spleen is increased in size in acute yellow atrophy of the liver, and is unchanged in yellow fever. The urine in yellow atrophy of the liver is acid throughout, and contains leucin and tyrosin, while as soon as jaundice appears in yellow fever the urine becomes alkaline. Yellow fever is ushered in with a chill, while in acute yellow atrophy of the liver it rarely begins with a chill. The pulse in severe forms of yellow fever is gaseous in character, and rarely over 110, while in acute yellow atrophy of the liver the pulse may reach 140 to 150 per minute.

The stools are dark and fluid in yellow fever, and firm and clay colored in acute atrophy of the liver.

Acute yellow atrophy of the liver is a rare disease occuring chiefly in pregnant women. It never occurs as an epidemic or endemic. Its causes are unknown.

The post mortem findings are, liver very much atrophied, sometimes to two-thirds of its normal size. It is so soft that it falls on itself. The capsule is loose, freely movable, very much wrinkled and opaque, or yellowish in appearance. The parenchyma is soft, flabby and brittle, and varies in color from a bright red to a yellow red.

On section, the outlines of the lobules are lost and only a detritus of granular matter is left. The blood is darker and thicker than normal and coagulates imperfectly. The heart is jaundiced, fatty and pultacious. The spleen is enlarged and softened. The kidneys are slightly enlarged and in most cases in a state of acute fatty degeneration. Hemorrhages from the mucous membrane of the stomach and intestines are common.

It is not difficult to diagnose pernicious malarial fever and acute yellow atrophy of the liver from yellow fever; it is, however, very difficult to differentiate between the mild form of yellow fever and dengue fever.

